# The electron donating capacity of biochar is dramatically underestimated

**DOI:** 10.1038/srep32870

**Published:** 2016-09-15

**Authors:** Antonin Prévoteau, Frederik Ronsse, Inés Cid, Pascal Boeckx, Korneel Rabaey

**Affiliations:** 1 Center for Microbial Ecology and Technology, Ghent University, Coupure Links 653, 9000 Ghent, Belgium; 2 Department of Biosystems Engineering, Ghent University, Coupure Links 653, 9000 Ghent, Belgium; 3 Laboratory of Applied Physical Chemistry, Ghent University, Coupure Links 653, 9000 Ghent, Belgium

## Abstract

Biochars have gathered considerable interest for agronomic and engineering applications. In addition to their high sorption ability, biochars have been shown to accept or donate considerable amounts of electrons to/from their environment via abiotic or microbial processes. Here, we measured the electron accepting (EAC) and electron donating (EDC) capacities of wood-based biochars pyrolyzed at three different highest treatment temperatures (HTTs: 400, 500, 600 °C) via hydrodynamic electrochemical techniques using a rotating disc electrode. EACs and EDCs varied with HTT in accordance with a previous report with a maximal EAC at 500 °C (0.4 mmol(e^−^).g_char_^−1^) and a large decrease of EDC with HTT. However, while we monitored similar EAC values than in the preceding study, we show that the EDCs have been underestimated by at least 1 order of magnitude, up to 7 mmol(e^−^).g_char_^−1^ for a HTT of 400 °C. We attribute this existing underestimation to unnoticed slow kinetics of electron transfer from biochars to the dissolved redox mediators used in the monitoring. The EDC of other soil organic constituents such as humic substances may also have been underestimated. These results imply that the redox properties of biochars may have a much bigger impact on soil biogeochemical processes than previously conjectured.

Biochars are the solid, recalcitrant organic carbon compounds derived from biomass pyrolysis of a variety of feedstock sources in an O_2_ depleted atmosphere. Biochars are generally porous, carbon-rich, and their physicochemical properties can strongly vary with the choice of feedstock and the parameters of the pyrolysis treatment. It has recently attracted a great deal of interest for its possible use in agronomic and engineering applications^1^,^2^. Biochars can improve soil fertility^3^,^4^, promote carbon sequestration^5^,^6^,^7^, favor contaminants removal^8^, and mitigate soil greenhouse gas emission such as CO_2_, CH_4_ and N_2_O^9^,^10^. The main assumption for the beneficial properties of biochars has been their large specific surface area and corresponding surface chemistry leading to extensive sorption capacity for crop nutrients, pollutants or gases^2^,^8^,^11^,^12^,^13^,^14^,^15^,^16^,^17^,^18^. The redox properties of chars has only been very recently studied and proposed as a possible cause for numerous (bio/geo)chemical processes^1^,^19^,^20^,^21^,^22^,^23^,^24^,^25^,^26^,^27^,^28^. These studies proved that biochars from various feedstock sources can either accept, donate or mediate substantial amounts of electrons in their environment, either abiotically or in relation with microorganisms. Mediated electrochemical measurements were used by Klüpfel *et al*. to assess the electron accepting capacity (EAC) and electron donating capacity (EDC) of wood-based and grass-based biochars^20^. The EAC is defined as the maximal amount of electrons that can accept a specific char from a sufficiently reductive solution. Oppositely, the EDC is the maximal amount of electrons that can provide a char to a sufficiently oxidative solution. The sum of EAC and EDC is defined as the electron exchange capacity (EEC). These capacities are related to the amount and nature of electron accepting moieties (likely quinones and polycondensed aromatic structures) and electron donating moieties (likely phenolic compounds) within the char. These redox moieties are transformation products derived from the pyrolysis of lignin and cellulose. As such, pyrolysis parameters, and especially the highest treatment temperature (HTT), control the magnitude of EECs^20^. Klüpfel *et al*. showed that the EAC of wood-based biochar reached a maximum of 0.54 mmol(e^−^).g_char_^−1^ for a HTT of 500 °C. The EDC apparently reached a maximum for a HTT of 400 °C (0.2 mmol.g^−1^) before decreasing dramatically for HTT ≥ 500 °C (≤0.03 mmol.g^−1^)^20^.

The potential for electron donation by biochar was demonstrated with a biochar from stillage residue pyrolyzed at 600 °C, which removed Ag^+^ by adsorption and reduction to Ag nanoparticles^25^ up to an equivalent of 0.83 mmol(e^−^).g_char_^−1^. This is much higher than the EDC previously measured for wood (0.03 mmol.g^−1^) or grass-based biochars (0.10 mmol.g^−1^) with identical HTT^20^. Unfortunately, the ratio of Ag^+^ either reduced or only adsorbed under cationic form could not be quantified. Another report showed that the bacterium *Geobacter metallireducens* was able to perform either denitrification (i.e. a reduction) or acetate oxidation with wood-based biochar (HTT: 550 °C) as the only electron donor or acceptor, respectively^22^. The bacteria were not expected to oxidize (or reduce) all the redox moieties “chemically available” within the char because of their restricted access in char pores as well as the thermodynamic limits of their metabolism. The authors were therefore surprised to find that their “bioavailable” EEC (0.86 ± 0.01 mmol(e^−^).g_char_^−1^) was even higher than the chemically determined EECs measured by Klüpfel *et al*. for similar wood-based biochars produced at similar HTTs (0.59 ± 0.09 mmol.g^−1^ and 0.18 ± 0.2 mmol.g^−1^ for 500 °C and 600 °C HTT, respectively)^20^.

The redox properties of biochars could be substantially involved in numerous biogeochemical phenomena such as, e.g., N_2_O mitigation^21^. Regarding the limited amount of data related to EEC values of biochars as well as some apparent discrepancies, we aimed here to quantify the amount of electrons that can be accepted or donated by wood-based chars pyrolyzed at different HTTs. To do so, we took advantages of highly accurate hydrodynamic electrochemical techniques with rotating disc electrodes (RDE) in order to monitor the redox state of oxidative or reductive solutions reacting with the biochars^29^. After validation of the method, we compared our values with previously published data obtained with similar biochars (feedstock from identical genus – *Pinus* – and identical HTTs). We showed that the EDC of biochars has so far largely been underestimated, very likely because a very slow rate of electron donation from biochars to redox solutions is quickly reached and could have been unnoticed. We observed a similar phenomenon for grass-based biochars as well as synthetic lignin powder and synthetic humic acid. We review the putative reasons for this sluggish electron transfer. Finally, we discuss the need for further investigations of these redox mechanisms and their possible implications for biochar-mediated soil biogeochemical processes.

## Results and Discussion

### Electrochemical analysis for EDC determination

Three sets of char were prepared at different HTTs (400, 500 or 600 °C) and designated in this study as ‘char-400’, ‘char-500’ and ‘char-600’ accordingly. The ground chars reacted for different times in abiotic, oxidative aqueous solution (buffered 50 mM ferricyanide, pH 6.5) under mixing before electrochemical analysis. All procedures are detailed in the Method section.

**Figure 1:**
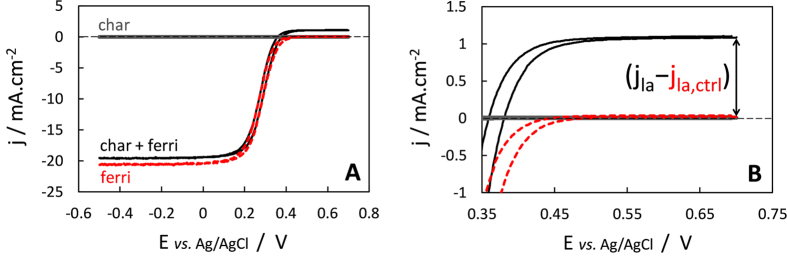
(**A**) Cyclic voltammograms recorded at t = 20 d in a suspension of char-400 in 50 mM ferricyanide, 0.1 M PB, 3 M NaCl (“char + ferri”, black curve); in controls in the absence of char (“ferri”, red dotted curve) or in the absence of ferricyanoide (“char”, grey curve). Recorded at a scan rate of 50 mV.s^−1^ and an electrode rotation speed of 1000 rpm. (**B**) Zoom on the anodic plateaus corresponding to the mass transfer limiting current density j_la_ for ferrocyanide oxidation. The amount of electrons donated by the char is proportional to (j_la_−j_la,ctrl_), difference between the anodic plateau currents recorded in the presence of char and in the control without char.

Hydrodynamic cyclic voltammograms (CVs) show that the 50 mM ferricyanide solution oxidized redox moieties from the char-400 in suspension for 20 days (Fig. 1). While the control devoid of char (red, dotted curve) showed an almost zero anodic plateau current (j_la,ctrl_, 20 μA.cm^−2^), the presence of 35.0 mg char-400 increased j_la_ to 1080 μA.cm^−2^ (black curve). The amount of electrons donated by the char (ED_m_) is proportional to (j_la_ − j_la,ctrl_) and was 4.8 mmol(e^−^).g_char_^−1^ (see Methods and equations (9) and (10)). The presence of char did not modify the sigmoidal shape of the CV nor the difference between the anodic and cathodic plateaus currents. This proves that the total amount of dissolved [ferricyanide + ferrocyanide] stayed constant over the experiment^29^. In particular, it demonstrates the absence of notable adsorption of these redox species on the char surface. When the char suspension was devoid of ferricyanide (grey curve), the CV was flat with non-significant current over the full range of potentials (− 0.5 V to +0.7 V vs. Ag/AgCl). Typical redox moieties from chars have formal potentials (pH 7) ranging from − 0.5 V to 0 V for electron accepting quinones^30^, and +0.2 V to above +0.6 V for electron donating phenolic compounds^31^. The absence of peaks or plateaus on the CV proves that no significant amounts of these redox moieties from char-400 were dissolved, and that they were tightly bound to the char. This is in good agreement with studies showing that soluble organic carbon content from biochar drastically decreases with increasing HTTs and becomes quasi-nil for HTT ≥ 400 °C^14^,^18^,^32^,^33^.

If the CV measurement allows the aforementioned controls, the ED_m_ of chars was generally determined faster and even more accurately via chronoamperometry (C.A., see Supplementary Fig. S1).

### Amount of electrons donated over time

**Figure 2:**
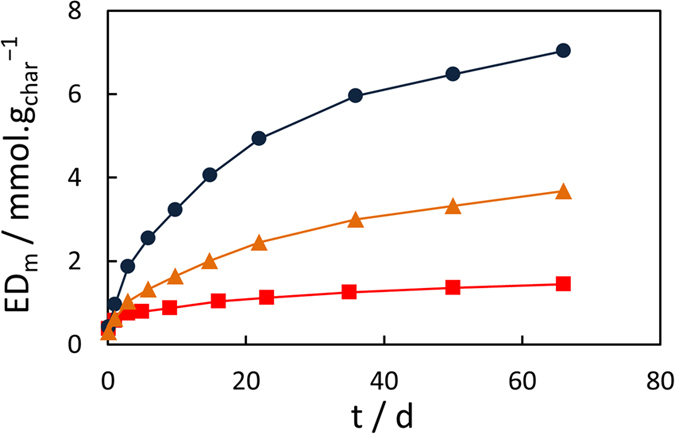
Electrons donated over time by char-400 (blue circles), char-500 (orange triangles) and char-600 (red squares). Error bars representing two standard deviations are too small to be visible (n = 2). For comparison with controls, the evolution of j_la_ values for char suspensions and solutions devoid of char are presented in Supplementary Fig. S2.

**Figure 3:**
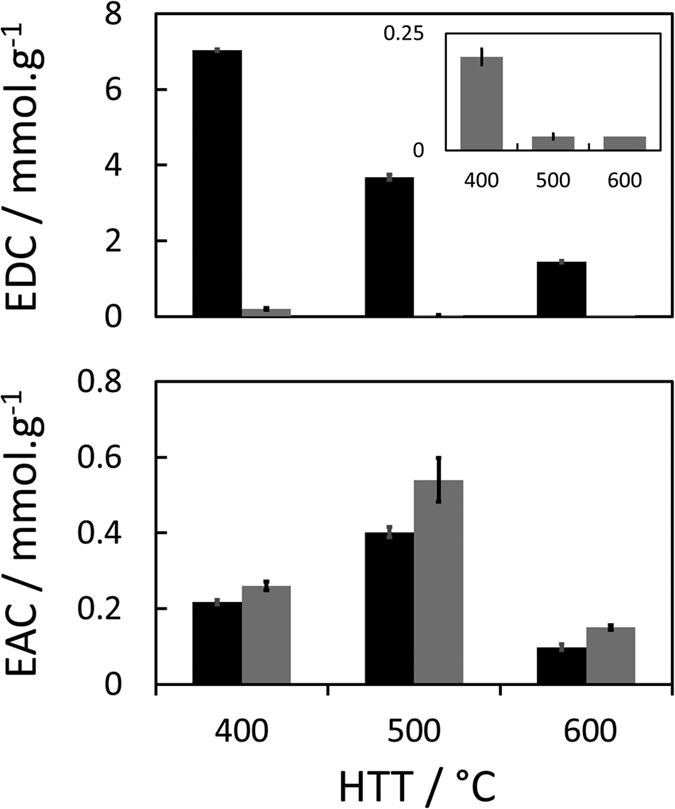
EDC (top) and EAC (bottom) of pinewood-based biochars pyrolysed at different HTTs. Our data (black bars, t = 66 d) are compared with those of Klüpfel *et al*. (grey bars, with a zoom in inset for their EDC). Error bars represent 2 standard errors (n = 3 for all results except our EDC where n = 2). Note the 10-times difference in scale between the EDC and EAC charts.

The kinetics of electron donation for the three chars are presented in Fig. 2. Electron transfer rates from chars were initially high (average of ~0.1 mmol(e^−^).g_char_^−1^.h^−1^ for the three first hours of reaction) and decreased very sharply over time (Supplementary Fig. S3). The ED_m_ of chars showed a continuous and drastic decrease with the HTT from 400 °C to 600 °C, as previously observed in Klüpfel *et al*.^20^. However, no redox equilibria were reached after 66 days of reaction under our conditions, whereas Klüpfel *et al*. assumed that apparent equilibria were achieved for all chars after only ~1 h. In addition, the differences in EDCs between both studies are particularly large for similar pinewood-derived biochars. We observed between one and two orders of magnitude higher EDCs (t = 66 d) for char-400 (7.0 mmol(e^−^).g_char_^−1^ vs. 0.20 mmol.g^−1^ in Klüpfel *et al*.), char-500 (3.7 mmol.g^−1^ vs. 0.03 mmol.g^−1^) and char-600 (1.4 mmol.g^−1^ vs. 0.03 mmol.g^−1^). Furthermore, the fact that the equilibria were not completely reached (only pseudo-plateaus reached on Fig. 2) indicates a certain underestimation of our own values of EDCs (which is also demonstrated by the substantial impact of initial ferricyanide concentration on the kinetics and pseudo-plateau values, see Supplementary Fig. S4). The minor difference in the pinewood-char preparation (30 min charring at HTT vs. 60 min for Klüpfel *et al*.) would not explain such differences in EDCs considering that char particles sizes (≤50 μm) and elemental compositions of the chars produced at identical HTT were similar in both studies (see Table 1 in Methods section for our values). Respective EDC values are compared on Fig. 3. Most redox moieties in the char are believed to contain oxygen^20^. The EDC increased almost linearly with the elemental oxygen content of the chars (Supplementary Fig. S5). The average ratio of electron donated per O slightly decrease with HTT from 0.71 (char-400) to 0.43 (char-600), while it was calculated to be between 0.003 and 0.015 for the similar biochars of Klüpfel *et al*.^20^. A probable reason for these discrepancies and another comparative experiment will be presented further. The decrease of EDC with HTT is very likely associated with the decrease in the abundance of oxygen functional groups (e.g. hydroxyl or ether) with charring temperature (see Table 1)^14^,^20^. In particular, the very large decrease in EDC between char-400 and char-600 could be related to a transition point at HTT between 450 °C–550 °C with the onset of aromatization coupled with a drastic decrease in hydroxyl functions due to the dehydration of lignin-derived phenols and alcohols^34^. The increasing aromatization of the chars with increasing HTTs was also confirmed by the continuous rise of their aromatization index (AI) from 0.63 for char-400 to 0.83 for char-600 (see Table 1). Wood-based biochars from Klüpfel *et al*. presented similar increasing trend and values of AI from 0.52 (char-400) to 0.80 (char-600).

### Controls

**Figure 4:**
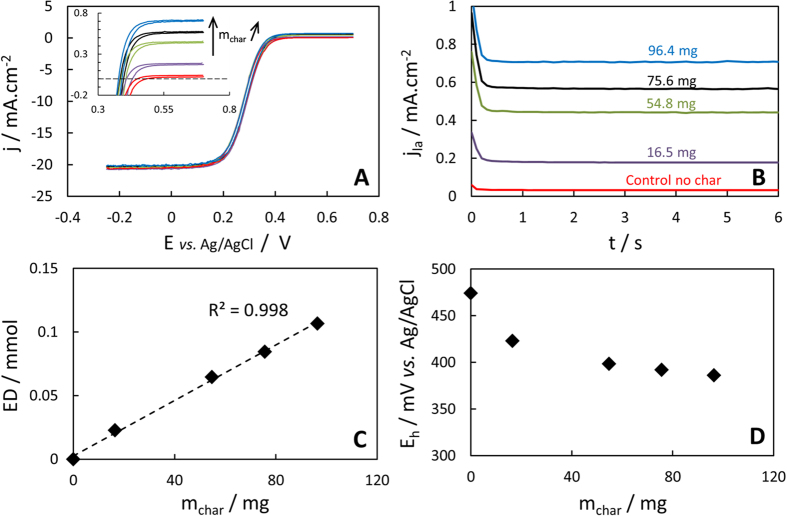
Impact of the mass of char-600 initially suspended in the oxidative solution (all data for t = 30 d). (**A**) CVs of char-600 suspensions of different masses of char (control devoid of char in red); 1000 rpm, 50 mV.s^−1^; the inset presents the zoom on anodic plateaus. (**B**) Corresponding CAs recorded at +0.7 V vs. Ag/AgCl and 1000 rpm. Respective masses of char-600 are stated on the chart. (**C**) Linearity of the amount of electrons donated (ED) and the mass of char initially suspended. R^2^ is the coefficient of determination for a simple linear regression. (**D**) Final E_h_ measured at open circuit potential, 1000 rpm.

Figure 4A shows the CVs recorded in redox suspensions of different mass of char-600 (t = 30 d). Conservation of the difference between anodic and cathodic current plateaus of CVs for each mass of char suspended (and control) confirmed the absence of noticeable adsorption of ferri- or ferrocyanide on the char surface (*vide supra*). Even for the maximum mass of char (96.4 mg), the final fraction of ferricyanide which has been reduced to ferrocyanide was only 3%, which indicates only a limited decrease in the oxidative power of the redox solution. Corresponding CAs recorded at +0.7 V (Fig. 4B) confirmed and refined the values of the anodic plateau currents j_la_ to access the amount of electrons donated by the char (ED). Figure 4C shows that the amount of electrons donated after 30 days was quasi-proportional to the mass of char-600 suspended. The final redox potentials (E_h_) of the char-600 suspensions are plotted in Fig. 4D. E_h_ value is determined by the ratio [ferri-]/[ferrocyanide] and follows the Nernst equation. The decrease of E_h_ with the mass of char initially suspended reflects the increasing amount of ferricyanide reduced and therefore a decrease of the oxidative power of the solution over time (and for larger mass of char). The good linearity of ED with the mass of char strongly suggests that the redox solution was sufficiently oxidative (i.e. concentrated in ferricyanide) to oxidize most of the oxidable redox moieties of the char. Still, a slight decrease in the EDC with the mass of char introduced indicates that minor thermodynamic and/or kinetics limitations of the redox process may have led to a slight underestimation of our EDC values (Supplementary Fig. 6). For all char suspensions from Fig. 2 (~30 mg char), the E_h_ of the solutions typically evolved from +0.48 V initially to ~+0.4 V vs. Ag/AgCl at the end of the experiment. It is noteworthy that this range of E_h_ is higher or ~ equal to the constant E_h_ at which Klüpfel *et al*. kept their redox solution to perform EDC measurements (+0.405 V vs. Ag/AgCl). This means that solutions in both studies had similar oxidative power, thermodynamically speaking.

The proportional relationship between the mass of char and ED (Fig. 4C) is expected if only the redox moieties of the chars are responsible for the reduction of ferricyanide. Still, all controls were performed to discard any (improbable) ferricyanide reduction by other chemical entities present. The chloride ion (used as supporting electrolyte) is a particularly weak reductant and did not reduce ferricyanide, even in the presence of char (absence of any putative catalytic effect of the latter), as shown on Supplementary Fig. S7. Water was not significantly reducing ferricyanide since its oxidation would necessarily produce some O_2_, which was not the case (Supplementary Fig. S8). Even the nature of the container surface itself (polypropylene) was tested and not involved in any electron transfer (Supplementary Fig. S9).

Finally, we showed that even for the less stable of the three chars (lowest HTT char-400)^35^, the high EDC measured was not due to an hypothetical accelerated rate of biochar carbon mineralization to CO_2_ because of ferricyanide (Supplementary Fig. S8 and Method S1). All these controls confirms that the high EDCs measured were indeed related to the electron donating moieties from the chars, such as e.g. phenolic compounds^20^.

### Electron accepting capacities

EAC initially increased with HTT and was maximal for char-500 (0.40 ± 0.02 mmol(e^−^).g_char_^−1^) before sharply decreasing for char-600 (0.10 ± 0.01 mmol.g^−1^). The linearity of the amount of electrons accepted with the mass of char-500 and corresponding raw data (CAs) are presented in Supplementary Fig. S10. Contrary to EDC, EAC values as well as the trend of EAC variation with HTT are in excellent agreement with the data from Klüpfel *et al*.^20^, as illustrated on Fig. 3, and despite the use of different dissolved electron donors (NR in the present study while they used a zwitterionic viologen). This tends to prove that both methods were adequate for EAC monitoring. A very large difference with our slow EDC kinetics is that the redox equilibria for EAC measurements were reached in less than 4 days (no measurement was made at a shorter time). The putative reasons for this difference in kinetics will be discussed in the section “Possible reasons for sluggish electron donation”. Contrary to EDC, the EAC did not increased monotonically with the elemental O content in chars but showed a maximum for char-500 (Supplementary Fig. S5). The trend of EAC with HTT is probably due to the generation of electron-accepting quinone moieties up to 400–500 °C followed by their consumption for higher HTTs^20^. A fraction of EAC is also believed to be due to polycondensed aromatic structures newly formed for HTT ≥ 600 °C^20^.

### Discrepancy between EDC values

We determined EDCs via a different method than the one conducted by Klüpfel *et al*. While we punctually measured ferrocyanide concentrations over 66 days to obtain ED_m_, Klüpfel *et al*. monitored in real time the electron donation from the char to a working electrode using the dissolved oxidant as a redox mediator (radical form of 2,2′-azino-bis(3-ethylbenzothiazoline-6-sulphonic acid), ABTS^•−^, E^0′^ = 0.465 V vs. Ag/AgCl^36^)^20^. In that way the electrons supplied by a certain amount of char were transferred to the electrode and the EDC was obtained by integrating a baseline subtracted current of a CA over ~1 h (chronocoulometry). A good redox mediator must be stable in aqueous solution for the time scale of the experiment^37^ and ABTS is a well-established colorimetric mediator for fast processes (s to min) such as enzyme assays^38^. However the radical anion ABTS^•−^ can lack long term stability and is slowly reduced by water^39^,^40^. We attempted to perform EDC measurements with 1 mM ABTS^•−^ rather than 50 mM ferricyanide and observed that 89% of ABTS^•−^ had been reduced to ABTS^2−^ in the control devoid of char after 6 days (Supplementary Fig. S11). In comparison, only 0.065% of the stable ferricyanide had been reduced for the same delay in the absence of char. For periods longer than 2 days, the remaining ABTS^•−^ was reduced faster by water only (control) than in the char suspensions and the “apparent” ED_m_ of chars were therefore decreasing over time. EDCs of chars were consequently underestimated with our method when using ABTS^•−^. Still, the “apparent” EDC values we obtained with ABTS^•−^ (i.e. the maximum ED_m_ reached for t ≥ 20 h) were between 3-times and 14-times higher than the values from Klüpfel *et al*. Interestingly, our ED_m_ values after only 1 h of reaction with ABTS^•−^ were very similar to their EDC values, recorded for ~ 1 h of reaction at which they assumed to have reached at redox equilibrium (Supplementary Fig. S12)^20^.

In a similar method to Klüpfel *et al*. to monitor the EDC of humic substances with ABTS^•−^, no measurements were performed at working electrode potential above +0.52 V vs. Ag/AgCl because this led to a baseline current above 100 μA, at least partially because of ABTS^•−^ instability (i.e. homogeneous reduction by water followed by heterogeneous oxidation on the working electrode)^41^. Typical baseline current values were not provided in Klüpfel *et al*.^20^, but it can be speculated that the low CA potential (+0.405 V vs. Ag/AgCl, where only ~10% of ABTS is in its radical form ABTS^•−^ and able to oxidize the chars) was chosen to avoid this excessive baseline current. With their direct measurement and after the initial fast rate of electron donation from the chars (>1 h), it could be challenging to distinguish between a low, almost constant rate of electron donation and a potentially higher, constant baseline current of ABTS re-oxidation. Furthermore, integration of the unsubstantial difference would very likely lack accuracy. This is also strongly suggested on Supplementary Fig. S13 where we present our own continuous measurement of initial electron donation from char-500 to ferricyanide as if recorded in a system as used by Klüpfel *et al*.^20^. We therefore believe that the EDCs values provided in ref. [20] may have been underestimated because of an inadequacy of the method and mediator to notice and monitor the slow reaction of electron donation by the biochars.

We performed three RDE-ferricyanide-based measurements with grass-based biochars which also provided much higher EDCs than with continuous chronocoulometry with ABTS^•-^ (~10–15 mmol.g^−1^ vs. 0.25-0.7 mmol.g^−1^, respectively, see Supplementary Fig. S14).

The EDCs of compounds other than biochars have previously been assessed via identical or very similar ABTS^•−^ mediated measurements over short times (~1 h)^41^. Humic substances show numerous chemical and structural similarities with biochar^42^. The EDC of a synthetic humic acid (Aldrich) was previously measured at 1.6 mmol.g^−1^ 41. We assessed a 3-time higher EDC (5.0 ± 0.2 mmol.g^−1^, n = 3) for the same humic acid with our method after 100 days of reaction (Supplementary Fig. S15). Similarly, our EDC for synthetic lignin powder was much higher (15.2 mmol.g^−1^ after 15 days, Supplementary Fig. S16) than the one previously monitored in ~1 h with ABTS^•−^ (4.3 mmol.g^−1^)^20^. Batch-to-batch variation from the same supplier could explain a relative difference in EDC values of these commercial compounds. However, the 3-times differences in EDCs monitored with both techniques as well as the much longer redox reaction strongly suggest that a slow release of electrons from these compounds has also been unnoticed after 1 h of reaction with the continuous, ABTS^•−^-based method.

One may claim that comparison of results from different methods could be biased, however, we strongly believe that EDC and EAC values should be intrinsic properties of specific compounds and therefore not be substantially method-dependent, similarly than for e.g. specific surface area, cation exchange capacity, elemental composition, etc. A standardized method of determination should be developed to record accurate values and allow relevant comparisons between compounds. Before all, a clear definition of “EDC” itself should be discussed. Should the choice of a certain EDC value be determined by a certain time of reaction in a specific solution (concentration of the redox compound and redox potential)? Should the EDC include a putative fraction of electrons donated by mineralization or via irreversible oxidative polymerization (*vide infra*)? Should it correspond to the amount of electrons that the char can only donate in environmentally relevant conditions (and then which one)?

### Possible reasons for sluggish electron donation

Wood-based biochars are relatively hydrophobic and a fraction of the char particles can occupy the gas/liquid interface of a static solution. The addition of 1 mM cetyltrimethylammonium bromide (CTAB, surfactant enhancing porous carbon wettability)^43^ did not impact the electron donation kinetics of the most hydrophobic biochar (char-600, Supplementary Fig. S17). This proves that under our mixing conditions, a hypothetical wettability issue was not involved in the slow kinetics. Since CTAB is an effective antiseptic^44^, it also confirms that no (unexpected) microbial process was involved in the redox process.

The surface of biochars is negatively charged at circumneutral pH^18^. Pore sizes of chars range over several orders of magnitude but smaller ones are typically at the nanometer level^1^,^13^. This is also the typical scale of the electrical double layer thickness where mostly cations can reach the close vicinity of the negative surface^45^. An electrostatic repulsion could therefore restrain the penetration of dissolved anions in small pores, and in particular for the ferricyanide trianion. Porosity and pore accessibility quickly increase with HTT^18^,^19^, while the surface charge density decreases with HTT^1^. Despite having the lowest EDC, the char-600 showed a faster initial electron donation than the chars prepared at lower HTT (see Supplementary Fig. S18). The ferricyanide molecules very likely encounter an easier/faster access to the oxidizable moieties present in the pores of the char-600 than in the less accessible, more negatively-charged pores of lower HTT chars. We also showed that the redox equilibrium was reached much faster with the uncharged^46^ NR (for EAC) than with the ferricyanide trianion (for EDCs). All these results would initially favor the assumption of electrostatic repulsion of ferrocyanide limiting the kinetics within the pores of the chars. However, Klüpfel *et al*. recorded similar EAC values with a negatively charged zwitterionic viologen in only 1 h^20^, which rather thwart the hypothesis of an electrostatic impact. Finally, the relatively slow kinetics observed for the non or less porous lignin and humic acid powders (Supplementary Figs S15 and S16) also favor another hypothesis. A possible electrostatic impact could still be assessed by testing neutral or positively charged oxidants to monitor EDCs.

A slow mineralization (i.e. oxidation) of biochar carbon to CO_2_ could account for the slow kinetics of electron donation observed, but only partially (see Supplementary Fig. S8 and associated Method S1: a maximum of 7% of the electrons donated by char-400 after 10 days could be the result of mineralization).

Last but not least, the sluggish reaction could be due to intrinsically slow kinetics of oxidation for some recalcitrant compounds or to sequential irreversible oxidations of some phenolic species. The reversible 1-electron oxidation of phenolic moieties produces phenoxy radicals. The latter can further perform slower complex, irreversible oxidative polymerizations^47^,^48^,^49^. This has been observed abiotically either with ferricyanide^50^ or ABTS^•−^ 51, or via enzymatic processes^52^. The first, reversible oxidation would allow a redox cycling of the char, conserving its “redox buffer” properties for its environment, as shown by Klüpfel *et al*.^20^. On the contrary, the irreversible oxidative polymerization would not allow further redox cycling and would decrease the EEC. Further investigations are needed to understand the slow kinetics of electron donation from the chars as well as their redox cyclability. It is also essential to assess to which extent these electrons can be donated in environmentally relevant conditions.

## Conclusion

Understanding and evaluating redox reactions in soil are of crucial importance. This work provides strong evidences that biochars may donate much more electrons to their environment than previously considered. It also shows that wood-based biochar can typically give one order of magnitude more electrons than they can accept, potentially favoring reductive reactions in the subsurface. Our results also suggest that the EDC of other organic or mineral compounds from soil may have been underestimated if their kinetics of electron donation is slow. In this case, punctual measurements with stable redox probes may be preferred to continuous ones if their detection limit in term of reaction rate is too high.

Given their much larger mineral content, the redox properties of biochars from other feedstock sources (e.g. manures or sewage sludge) should be carefully assessed. For example, a high content in Fe and/or Mn in the char may significantly impact the EECs and the kinetics at which electrons are exchanged with their environment. Due to the high chemical complexity of chars or humic substances, it may also be useful to assess the EDC of redox model molecules bound within the biochar structure after primary degradation of lignin during pyrolysis (e.g. phenolic and aromatic compounds). Valuable information could derive from their (chemical and biological) oxidation mechanisms, kinetics and products determination, as well as the possible occurrence and extent of these redox processes in environmental conditions. A key question is indeed how much electrons a biochar can actually donate to a specific biogeochemical environment with respect to the chemical EDC measured in laboratory studies where the oxidation rate and extent are attempted to be maximized. Furthermore, ageing of biochars very likely impacts their redox properties before and after their addition to soil. In particular, ageing of chars can modify their surface via precipitation or sorption of organic and mineral compounds to their surface^53^.

The EDC and EAC should also be accurately measured and taken into account when studying any redox process occurring in the presence of biochars. For example, several recent reports showed that biochars^27^,^54^ can promote microbial activity by acting as electron shuttle between two delocalized microbial species^54^ or between a bacterial strain and Fe(III) mineral^27^. On the other hand, Saquing *et al*. recently proved that biochars can substantially support microbial activity only via their ability to provide or harvest electrons within the microbial metabolic pathways (i.e. irrespectively to biochars conductivity)^22^. This stresses the need to assess which of these nonexclusive mechanisms (conductivity or EECs) is mostly involved in supporting microbial metabolism, especially for biochars pyrolyzed at relatively low HTT (<600 °C) where EECs are high but electrical conductivity unsubstantial^55^.

More generally, our results further emphasize the potentially large impact of biochars on soil redox processes, either microbial or abiotic. The nature and extent of these redox processes will depend on the char characteristics (EEC, porosity, surface chemistry, charge and area, etc., which mostly rely on feedstock choice and charring conditions) and its surrounding soil and climate environment (soil composition and morphology, pH, plant and microbial ecology, temperature, hygrometry, etc.). A better understanding of the numerous and complex redox interactions between well-defined biochars and specific biogeochemical environments is therefore crucial in order to design adequate biochars for targeted beneficial purposes.

## Methods

### Chemicals

Na_2_HPO_4_.2 H_2_O, NaH_2_PO_4_.2 H_2_O, NaCl and potassium ferricyanide were purchased from Carl Roth; potassium ferrocyanide, neutral red (NR) from Sigma. All chemicals had higher purity than 98% and were used without further purification. Humic acid (20 wt% ash) and lignin (alkali) were from Aldrich.

### Solutions

All solutions were made with deionized water (18.2 MΩ.cm), passed through a Milli-Q purification system (Millipore) and made anaerobic by N_2_ gas bubbling (~30 min) with subsequent storage (>2 h) before use in an anaerobic workstation (GP-Campus, Jacomex, TCPS NV, Rotselaar, Belgium) under a N_2_:CO_2_ (90:10, v/v) atmosphere. Solution for EDC determination was 50 mM potassium ferricyanide (E^0′^ = +0.29 V vs. Ag/AgCl), 3 M NaCl and 0.1 M sodium phosphate buffer (PB) at pH 6.5. At the end of the experiments, the char suspensions were all at pH 6.5 ± 0.1. Highly concentrated NaCl acts as the supporting electrolyte and avoids migration impact on ferricyanide mass transfer, which could bias electroanalytical measurements based on diffusion/convection (Levich equation). EAC was determined in a solution made of ~5 mM of reduced NR_RED_ (E^0′^ = −0.49 V vs. Ag/AgCl) in an identical electrolyte. The electrolysis step to reduce commercial NR before its use is described in Supplementary Method S3.

### Chars

Commercially available bark-free pinewood (Bemap Houtmeel BV, The Netherlands) was used as feedstock containing a mixture of different pine species, of which *Pinus sylvestris* was the predominant one. Further compositional data are available elsewhere^56^. The production of char was carried out under N_2_ in a slow pyrolysis retort, which consisted of a vertical, tubular, stainless steel reactor (*d* × *L* = 3.8 cm × 30 cm) heated by an electric furnace, full details on the setup and procedure are available elsewhere^57^. From 70 g to 100 g of ~6 mm wood chips were first loosely packed in the reactor. The reactor was heated at a rate of ~17 °C min^−1 ^until the HTT was reached and held constant for 30 min, after which the reactor was cooled at ambient temperature. Pyrolysis gases were continuously evacuated by continuous N_2_ flow. Elemental analysis (C, H, N) was performed on biochars in triplicate in an elemental analyser (FLASH 2000 Organic Elemental Analyser, Thermo Scientific) according to standard procedure^58^. The oxygen content was calculated by mass balance on the ash-free basis. The samples (3 mg) were dried overnight (105 °C) before analysis and 2,5-bis(5-tert-butyl-benzoxazol-2-yl)thiophene (BBOT) was used as a standard reference. The ash content in the chars was determined in triplicate according to standard procedure^59^ in a muffle furnace (AAF 11/3, Carbolite). BET specific surface area of biochars was determined via N_2_ adsorption at 77.4 K with a TriStar II surface area analyzer (Micromeritics). The samples were pretreated with a VacPrep Degasser (Micromeritics). Selected physicochemical properties of the chars are listed in Table 1.

### Redox reactions in char suspensions

All dry chars were manually ground with a ceramic mortar and pestle to increase accessibility of the redox solution to the pores and accelerate the electron exchange rate. Particles of ground chars smaller than 50 μm were selected via sieving (Retsch, Germany). Unless otherwise specified, ~30 mg of chars were weighed (0.1 mg accuracy) in 50 mL polypropylene tubes wrapped under aluminum foil to avoid light exposure (the latter increases the very slow natural reduction of ferricyanide, see Supplementary Fig. S20). The chars were exposed to vacuum for at least 1 h in the airlock of the anaerobic workstation to remove residual O_2_. Note that a longer vacuum exposure (or its absence) did not substantially modified the EDC obtained (Supplementary Fig. S21). All the following steps were performed inside the anaerobic workstation. The tubes were filled with 55 mL of anaerobic oxidative or reductive solutions (~0.55 g_char_.L^−1^), without substantial headspace to favor wetting of relatively hydrophobic chars. The tubes were sealed and immediately subject to continuous shaking at 100 rpm (orbital shaker, Phoenix Instrument), initiating the redox reactions between the redox moieties of the chars and dissolved oxidant or reducer (t = 0):


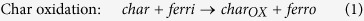



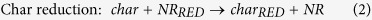


At any extent of reaction, the amount of electrons donated per mass of char (ED_m_ [mol(e^−^).g_char_^−1^]) is:


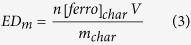


where n = 1 is the number of electrons exchanged per ferricyanide reduced, [ferro]_char_ the concentration of ferrocyanide generated from the ferricyanide reduction by the char [mol.cm^−3^], V the volume of solution (55 cm^3^) and m_char_ ([g]) the mass of char in suspension.

Similarly, the amount of electrons accepted per mass of char EA_m_ is:


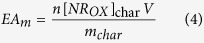


where n = 2 is the number of electrons exchanged per NR oxidized^60^ and [NR_OX_]_char_ the concentration of NR generated from NR_RED_ oxidation by the char.

### Electrochemical measurements for EDC determination

The measurements were performed in the char suspensions (or controls in the absence of char) using a potentiostat (VSP, Biologic, France) inside the anaerobic workstation at 30 °C. Glassy carbon RDEs (3 mm diameter (0.0707 cm^2^), A-011169, ALS, Japan) were used as working electrodes. They were successively polished on microcloth pads with 1 μm and 0.05 μm diameter alumina slurries (Buehler, USA), and a particle free pad, and thoroughly rinsed with distilled water after each step. No decrease of electroactivity toward ferri/ferrocyanide couple was detected over 20 successive measurements, whereas the RDE was re-polished before every measurement in NR solutions due to slow but significant decrease of electroactivity. A platinum spiral wire (10 cm) was used as counter electrode and all potentials in this manuscript are referred to a Ag/AgCl (3 M KCl) reference electrode (ALS, Japan, +0.205 V *vs.* SHE at 30 °C). The RDEs were rotated using an RRDE-3A rotator (ALS, Japan) in a 50 mL glass beaker.

Cyclic voltammetries (CVs) were performed at 50 mV.s^−1^ at a RDE rotation speed of 1000 rpm. Constant potential chronoamperometries (CAs) were periodically performed at 1000 rpm in the supernatants of the suspensions which were reintroduced in their respective tube after measurement. The potential was sufficiently high (+0.7 V vs. Ag/AgCl) to reach the anodic limiting current density j_la_ [A.cm^−2^] which is proportional to the total ferrocyanide concentration [ferro]_tot_ according to the Levich equation^45^:


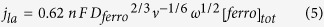



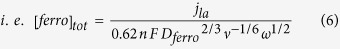


where F is the Faraday constant (96,485 C.mol^−1^), D_ferro_ the diffusion coefficient of ferrocyanide at 30 °C in our solution: 4.27 ± 0.28 cm^2^.s^−1^ (measured via Levich analysis, see Supplementary Fig. S22), ν is the kinematic viscosity of 3 M NaCl solution at 30 °C (9.83 × 10^−3 ^cm^2^.s^−1^)^61^, ω the RDE rotation speed (104.7 rad s^−1^) and [ferro]_tot_ in [mol.cm^−3^]. A small background current (j_la,ctrl_) is recorded in solutions devoid of char and subtracted to j_la_ in equation (6) to obtain the actual concentration of ferrocyanide generated by char oxidation [ferro]_char_:


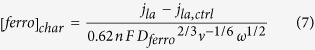


Finally ED_m_ is obtained by replacing [ferro]_char_ in equation (3):


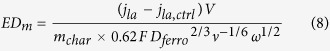



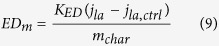


With K_ED_ the constant ratio between the charge donated by the char [mol(e^−^)] and the background-subtracted limiting current density (j_la_−j_la,ctrl_) in [A.cm^−2^]:


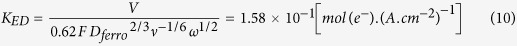


Finally, the EDC of biochars corresponds to the final ED_m_ once a (quasi-)equilibrium between the char and the sufficiently oxidative solution is reached.

### Electrochemical measurements for EAC determination

The method was similar than for EDC determination. EA_m_ is proportional to the absolute value of the background-subtracted cathodic limiting current density (j_lc_−j_lc,ctrl_) for NR reduction recorded at −0.65 V vs. Ag/AgCl (CVs available in Supplementary Fig. S19):


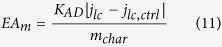


with


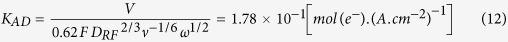


With D_NR_ the diffusion coefficient of NR at 30 °C in 3 M KCl. The latter was not retrieved from a database and was assumed to be 3.57 × 10^−6 ^cm^2^.s^−1^ (recalculated with the Stokes-Einstein equation from the diffusion coefficient at 25 °C of fluorescein in water (4.25 × 10^−6 ^cm^2^.s^−1^)^62^, which has a similar structure and molecular weight than neutral red). The EAC corresponds to the EA_m_ once an equilibrium between the char and the sufficiently reductive solution is reached. Experimental values in text are provided as the mean +**/−** standard deviation for n samples.

## Additional Information

**How to cite this article**: Prévoteau, A. *et al*. The electron donating capacity of biochar is dramatically underestimated. *Sci. Rep.* **6**, 32870; doi: 10.1038/srep32870 (2016).

## Supplementary Material

Supplementary Information

## Figures and Tables

**Table 1:** Physicochemical properties of pinewood feedstock and corresponding biochars pyrolyzed at different HTTs: pyrolysis yield (dry-basis); ash content and elemental composition of most common elements (both on dry basis); average carbon oxidation state (C_ox_); double-bond equivalent per carbon (DBE/C); aromaticity index (AI); specific surface area (SSA).

HTT	Yield	Ash	Elemental composition					(DBE/C)^c^	AI^c^	
			C	H	O	N	C_ox_^b^			
[° C]	[wt%]	[wt%]	[mmol(element).(g_char_)^−1^]				—	[mol DBE.(mol C)^−1^]		SSA [m^2^.g^−1^]
feedstock^a^	—	0.15	40.7	59.8	28.1	0.05	—	—	—	—
400	30.6	1.24	65.2	42.4	9.8	0.41	−0.35	0.69	0.63	1.65
500	24.8	1.15	69.8	34.7	7.1	0.29	−0.28	0.77	0.75	35.3
600	23.3	1.06	75.2	26.9	3.3	0.51	− 0.27	0.83	0.83	289

^a^Data from ref. 55; ^b^ and ^c^ calculated from elemental composition data according to refs 63 and 64, respectively.
