# Supplementary material for: The electron donating capacity of biochar is dramatically underestimated
Source: Sci Rep. 2016 Sep 15;6:32870. doi: 10.1038/srep32870 (PMC5024093; doi:10.1038/srep32870)
Supplement: Supplementary Information [file srep32870-s1.pdf]

Supplementary Information

The electron donating capacity of biochar is dramatically underestimated

Antonin Prévoteau, Frederik Ronsse, Inés Cid, Pascal Boeckx, Korneel Rabaey

Table of Contents:

Figure S1: Fast ED_m determination via chronoamperometry.......2
Figure S2: Evolution of j_{la} with time for suspensions of different chars and control.......3
Figure S3: Fast decrease of electron donation rates from the chars to the redox solution.......3
Figure S4: Impact of initial ferricyanide concentration on kinetics and apparent EDC.......4
Figure S5: Relation of EDC and EAC with elemental O content.......5
Figure S6: Slight impact of the mass of char on determined EDC.......5
Figure S7: Chloride does not reduce ferricyanide.......6
Figure S8: O_2 and CO_2 gas measurement.......7
Method S1: O_2 and CO_2 gas measurement, estimation of char mineralization.......7
Figure S9: Polypropylene does not reduce ferricyanide.......9
Figure S10: Impact of the mass of char on the amount of electron accepted EA.......10
Figure S11: "Apparent EDC" measurements with instable $ABTS^{\bullet-}$.......11
Figure S12: Comparison with published data after ~ 1 h of reaction.......12
Method S2: $ABTS^{\bullet-}$ production.......12
Figure S13: Our real-time data as if measured via 'mediated electrochemical oxidation'.......13
Figure S14: EDC measurements of grass-based biochars.......14
Figure S15: EDC measurements of synthetic humic acid.......15
Figure S16: EDC measurements of synthetic lignin powder.......16
Figure S17: Surfactant does not impact kinetics of electron donation.......16
Figure S18: Real-time measurements of initial electron donation from biochars.......17
Method S3: Solution for EAC determination.......18
Figure S19: CVs recorded in neutral red solution.......18
Figure S20: Stability of ferricyanide in solution / impact of light.......19
Figure S21: Impact of initial oxygen removal in recorded EDC values.......19
Figure S22: Levich model for ferrocyanide / determination of D_{ferro}.......20
Figure S22: Supplementary references.......20

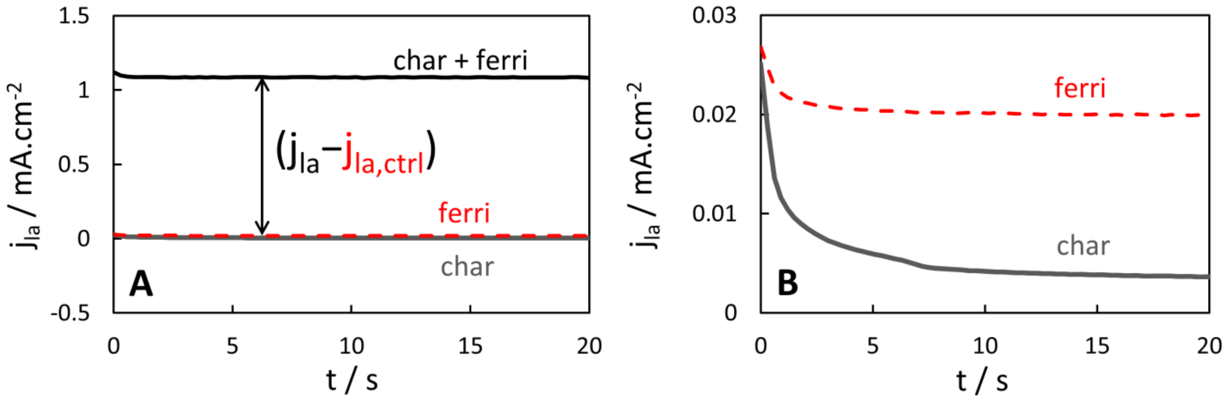

Supplementary Figure S1. (**A**) Chronoamperometry recorded in a suspension of char-400 in 50 mM ferricyanide, 0.1 M PB, 3 M NaCl ("char + ferri", black line); in the control in the absence of char ("ferri", red dotted line) or in the absence of ferricyanide ("char", grey line). Recorded at + 0.7 V vs. Ag/AgCl and 1000 rpm,30 °C, t = 20 d. (**B**) Zoom on CAs of the two controls. The current recorded in the ferricyanide solution devoid of char accounted for ~ 2 % of the current when the char was also in presence. "Steady-state" currents were reached after a couple of seconds when chars and ferricyanide are both in presence ("high" current), while it took up to 10-20 s for the smaller current recorded in the controls.

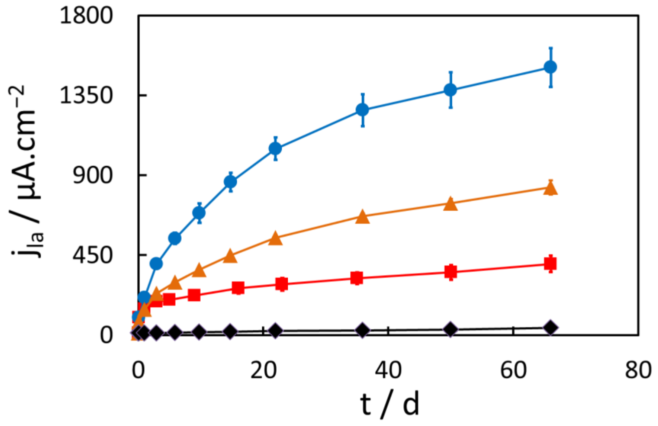

Supplementary Figure S2. Evolution of j_{la} with time for suspensions of char-400 (blue circles), char-500 (orange triangles), char-600 (red squares) and control (devoid of char, black diamonds). Error bars represent two standard deviations (n = 2). The error bars are sometimes visible since the masses of char initially introduced were slightly different for each duplicate. When the current density is normalized with respect to the mass of char introduced, error bars were too small to be visible such as in Fig. 2 (maximal relative standard deviation was 3 %).

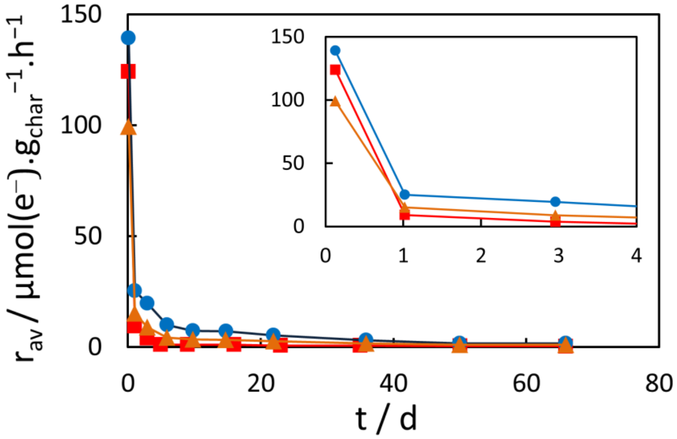

Supplementary Figure S3. Fast decrease of electron donation rates for the 3 chars (same color code than above). Each point represented at time t_n is the average electron donation rate between t_n and $t_{(n-1)}$ (i.e. the previous point). Inset: zoom on first days of reaction.

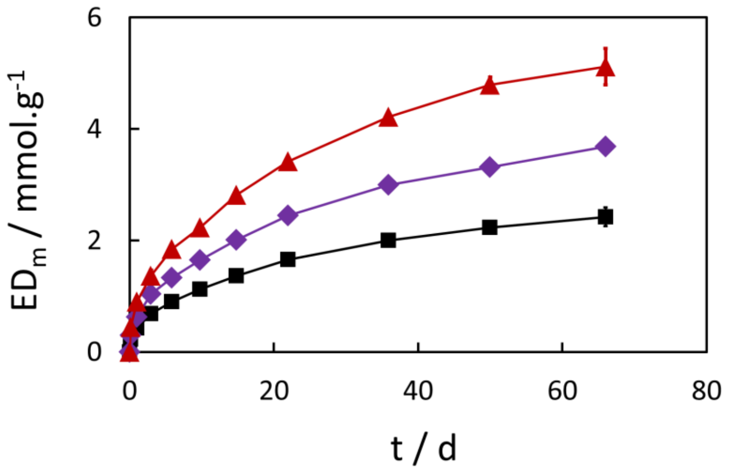

Supplementary Figure S4. Impact of ferricyanide concentration on the kinetics of electron donation from char-500. In solution of 0.1 M PB pH 6.5, 3 M NaCl and 10 mM ferricyanide (black squares), 50 mM (purple diamonds) and 250 mM (red triangles). Error bars represent 2 standard deviations (n =2).

These results show that the initial concentration of the oxidizing agent has a substantial impact on the kinetics of electron donation from the char and on the apparent thermodynamics (i.e. pseudo-plateau values and therefore EDC). The rate of the homogeneous redox reaction between phenolic compounds and ferricyanide, as well as the maximal extent of these reactions, have been shown to be decreasing with the [ferrocyanide] / [ferricyanide] ratio[1,2]. This is therefore probably the case for the oxidation phenolic compounds within the char, where smaller initial concentration of ferricyanide involves a faster increase of the aforementioned ratio. Note that this behavior was also observed for homogeneous redox reactions between ABTS$^{\bullet -}$ and phenolic compounds[3]. The most important outcome from these results is that the EDC values provided in the present study (recorded with 50 mM ferricyanide) are also underestimated to a certain extent.

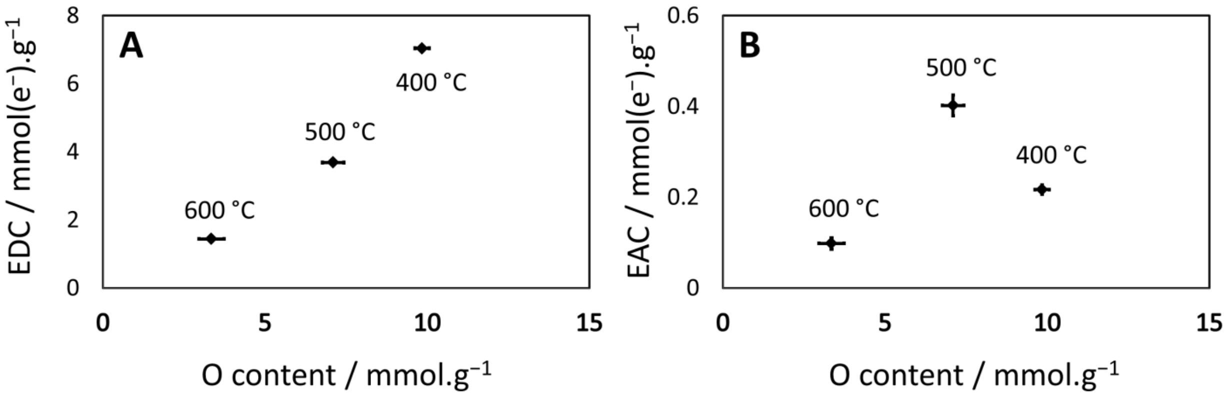

Supplementary Figure S5. Relation of EDCs (**A**) and EACs (**B**) with elemental O content for the 3 biochars. Respective HTTs are stated on the charts. Note the difference in scale between (**A**) and (**B**). Error bars represent 2 standard deviations for 2 (**A**) and 3 (**B**) samples, respectively.

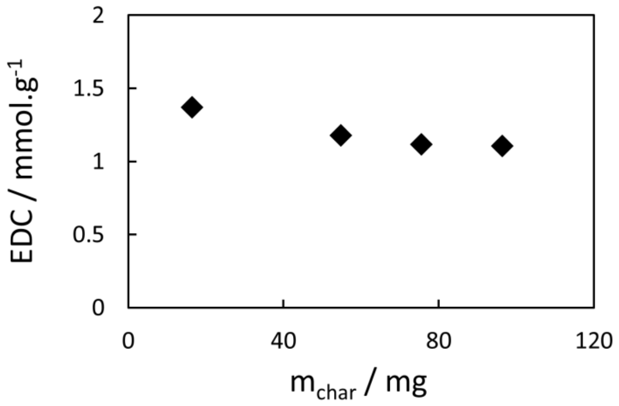

Supplementary Figure S6. EDC measured in suspensions of char-600 for increasing mass of char initially suspended. Recorded at t = 30 d. A slight decrease of the apparent EDC with the mass of char reacting is due to the slight decrease of the redox potential E_h (i.e. oxidative power) of the solution with the mass of char (i.e. amount of reaction) as shown in Fig. 3D. See main text for further discussion.

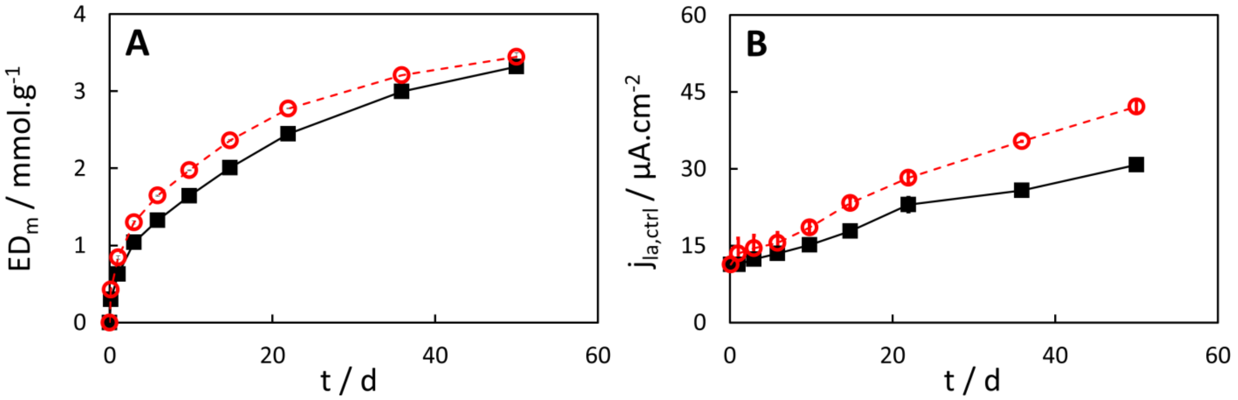

Supplementary Figure S7. (**A**) Electron donation over time of char-500 when the supporting electrolyte is 3 M NaCl (black square) or 3 M $NaNO_3$ (red circles). (**B**) Evolution of the anodic plateau current of the controls (devoid of char). Error bars represent 2 standard deviations (n = 2).

Note: the dynamic viscosity μ at 30 °C of 3 M $NaNO_3$ is slightly lower (1034 µPa.s)[4] than the one of 3 M NaCl (1080 µPa.s)[5], which induces a slightly higher D_{ferro} in 3 M $NaNO_3$ (4.6 cm^2.s^{-1}, measured by us via a Levich measurement, see above in S.I.). The latter value was taken into account for calculating the ED_m of the char in $NaNO_3$ via equation (8).

This results show that chloride ions are not reducing ferrocyanide (even in the presence of char, potential catalyst), in which case (an apparent) ED_m and/or $j_{la,ctrl}$ would be higher in the presence of Cl^-.

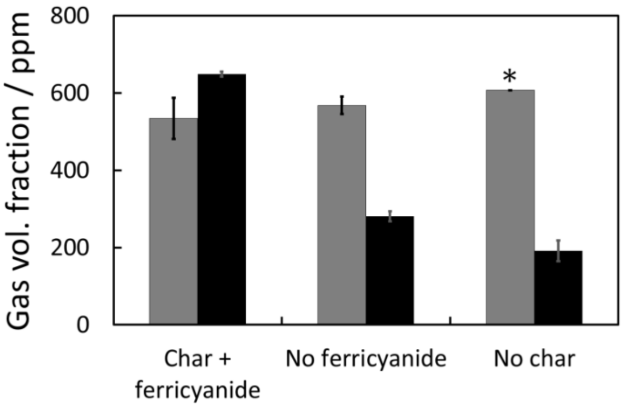

Supplementary Figure S8. Oxygen (grey) and CO_2 (black) gas measurements in headspace of penicillin bottles after 10 days of reaction of char-400 with 50 mM ferricyanide; controls without ferricyanide or without char. Error bars represent 2 standard deviations for 2 samples (except * where n = 1 due to a sample loss). Ferricyanide (either with or without char) was not significantly reduced by water since it would have produced an excess amount O_2 measurable in the headspace. The char may have been slightly mineralized to CO_2 especially in the presence of ferricyanide. However this possible mineralization could not explain the high EDC of char-400 (see calculations below).

Supplementary (associated) Method S1. Around 100 mg of degassed char-400 and 50 mL of anaerobic ferricyanide solution (see method in core text) were introduced in gas-tight 120 mL amber glass penicillin bottles flushed with N_2. Two controls (either without char or without ferricyanide) were also prepared in duplicate. All bottles were shaken at 120 rpm for 10 days before gas composition analysis of the 70 mL headspaces for O_2 an CO_2. The latter was performed with a Compact GC (Global Analyser Solutions, Breda, The Netherlands) equipped with a Molsieve 5A pre-column and Porabond column (O_2) and a Rt-Q-bond pre-column and column (CO_2). Concentrations of gases were determined by means of a thermal conductivity detector.

Associated calculation for char mineralization. We assumed that the CO_2 generated by char carbon oxidation was related to the difference between the CO_2 concentration measured with (char + ferricyanide) and without char; i.e. 457 ppm or 19 µM i.e. **1.33 µmol $CO_{2,gas}$** in the 70 mL headspace. Assuming gas-liquid equilibrium for CO_2 (with a dimensionless Henry solubility H^{cc} = 0.81 and an insignificant amount of H_2CO_3 form with respect to dissolved $CO_{2,aq}$)[6], there was 15.4 µM of $CO_{2,aq}$ deriving from char mineralization in 50 mL aqueous phase i.e. **0.77 µmol $CO_{2,aq}$**. Finally assuming acid-base equilibrium in solution between $CO_{2,aq}$ and HCO_3^- at the final pH 6.5 (pKa^* = 6.3), the char mineralization would have generated 9.7 µM i.e. **0.49 µmol HCO_3^-**.

Total carbon mineralized from char = 1.33 + 0.77 + 0.49 = 2.59 µmol(C) for 102 mg of char-400, i.e. **25.4 µmol(C).g_{char}^{-1}** or ~ 0.04 % of total char carbon initially introduced which was mineralized to CO_2.

In term of electrons potentially donated due to mineralization, assuming an average initial oxidation number for carbon in char-400, C_{ox} = – 0.35 (see **Table 1**) to be oxidized to CO_2 (C_{ox} = + 4):

25.4 µmol(C).g_{char}^{-1} × [4 − (− 0.35)] = **110 µmol(e^-).g_{char}^{-1} of the ED_m which could have been due to the char mineralization.**

The ED_m measured in the corresponding solution after 10 days was much higher at **1.49 mmol(e^-).g_{char}^{-1}**. In conclusion the mineralization of the char under oxidative conditions may have influenced the ED_m measured, but only to a minor extent.

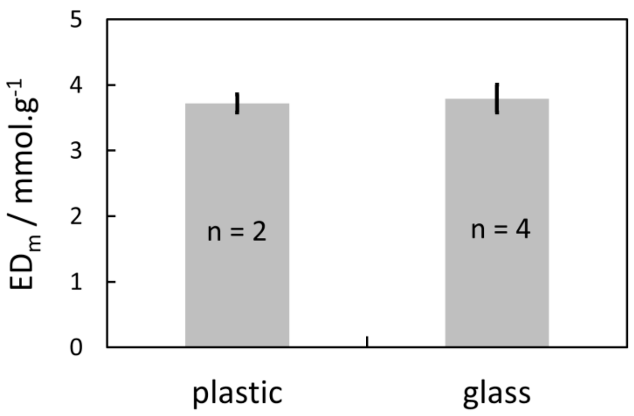

Supplementary Figure S9. The nature of the container material did not impact the amount of electrons donated by the char to the ferricyanide solution either with polypropylene (Falcon tubes 50 mL) or glass (penicillin bottles 50 mL). Data recorded after 12 days of reaction of char-400 with the ferricyanide solution. Error bars represent 2 standard deviations for n samples.

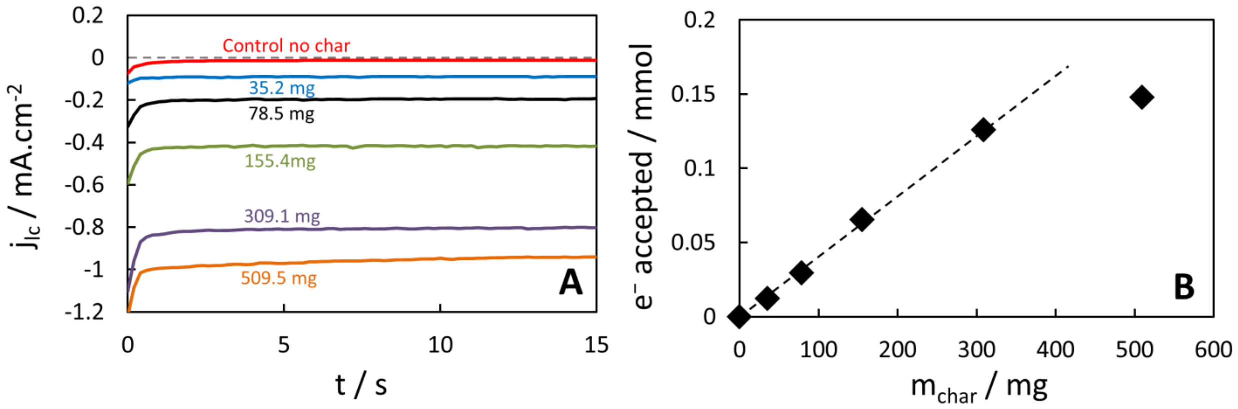

Supplementary Figure S10. Impact of the mass of char-500 on EAC measurement. (**A**) CAs recorded at − 0.65 V vs. Ag/AgCl and 1000 rpm in ~ 5 mM NR, 0.1 M PB pH 6.5 and 3 M NaCl. Respective mass of char-500 are stated on the chart. All cathodic limiting currents are reached ~ 1 s after the CA started. However, CAs recorded with the 2 highest masses of char-500 showed a slow decrease of the absolute value in current. Value of currents was therefore taken at t ~ 2 s to calculate the amounts of electrons accepted. (**B**) Proportional relation between the amount of electrons accepted by the char-500 and its mass initially introduced up to 310 mg. For 509.5 mg of char the NR solution was not sufficiently concentrated to reduce completely the char-500 (as much as ~ 30 % of NR had been reoxidized by the char). All measurements made after 4 days of reaction when the redox equilibria had been reached (stability of j_{pc}). No measurements were performed before these 4 days.

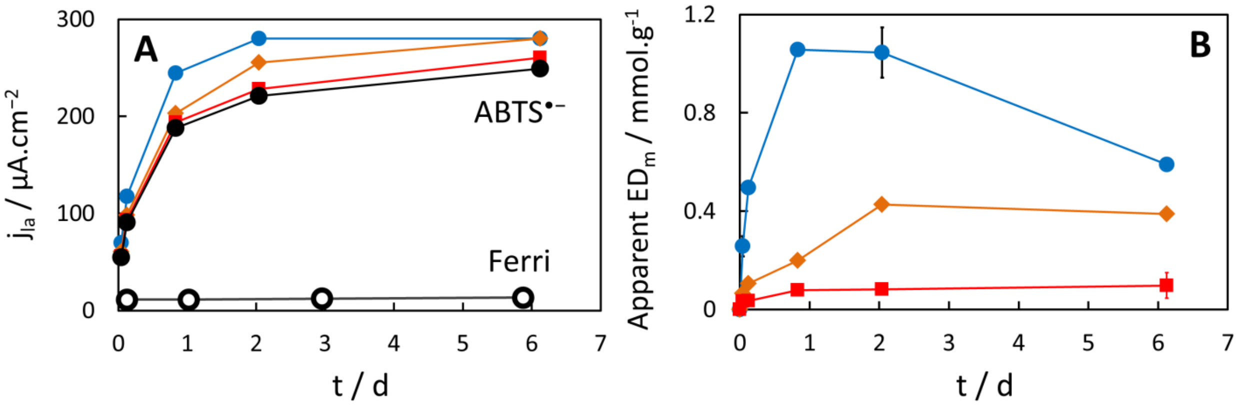

Supplementary Figure S11. Inadequacy of ABTS$^{\bullet-}$-mediated measurement for our method due to ABTS$^{\bullet-}$ long term instability (**A**) Evolution of $j_{la,\ ABTS}$ for suspensions of char-400 (blue circles), char-500 (orange triangles) and char-600 (red squares) in ~ 1 mM ABTS$^{\bullet-}$, 3 M NaCl, 0.1 M PB pH 6.5, 30 °C. The ABTS$^{\bullet-}$ control (without char, full black circles) was not stable and was naturally reduced in water. The 50 mM ferricyanide (empty black circle) solution was much more stable. No current could evolve higher than 280 µA.cm^{-2} since all ABTS$^{\bullet-}$ was reduced to ABTS^{2-} at that point. All char suspensions contained ~ 11 mg of char. (**B**) Evolution of the corresponding "apparent ED_m" over time (n = 2), proportional to ($j_{la,ABTS}$–$j_{la,ctrl,\ ABTS}$) as calculated with equation (8), assuming D_{ABTS} = 3.6 × 10^{-6} cm^2.s^{-1}. The apparent ED_m decreased once the rate of ABTS$^{\bullet-}$ reduction became higher in the control than in the char suspensions (i.e. when most ABTS$^{\bullet-}$ was already reduced in the latest). Most interestingly, the maximal apparent ED_m reached, which can only be an underestimation of the EDC, were still 5-times (char-400), 14-times (char-500) and 3-times (char-600) higher than the values recorded by Klüpfel et al. with ABTS$^{\bullet-}$ with short term (~ 1 h) measurements[7]. Finally, our ED_m values recorded after only 1 h of reaction with ABTS$^{\bullet-}$ were very similar to their EDC values recorded for ~ 1 h of reaction, at which they assumed to have reached at redox equilibrium (see Figure S12). All these results

strongly suggest that the underestimation of EDCs was indeed due to the slow kinetics of e^- transfer, unnoticed after ~ 1 h.

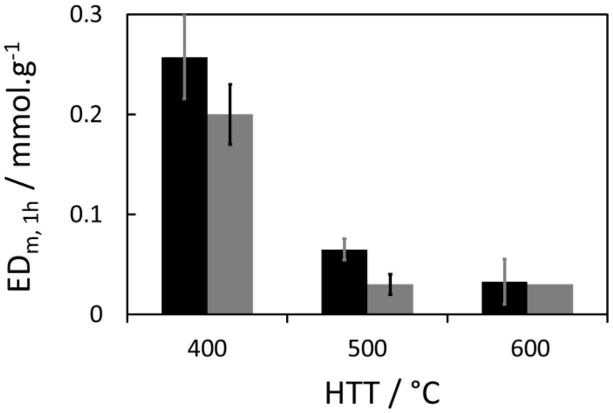

Supplementary Figure S12. Comparison of our ED_m values after 1h or reaction with $ABTS^{\bullet-}$ (black bars) and the values of EDC from Klüpfel at al. (with their total measurement time ~ 1 h, grey bars).[7] Error bars represent 2 standard deviations for n = 2 (black) and n = 3 (grey) samples.

Supplementary Method S2 for $ABTS^{\bullet-}$ production: $ABTS^{\bullet-}$ was produced by oxidation of $ABTS^{2-}$ in a similar electrolysis setup than previously described for NR reduction, but at a working electrode potential of + 0.7 V vs. Ag/AgCl, and with a cation exchange membrane (CMI-7000, Membranes International, Ringwood, USA) to separate both compartments. Once oxidized, the $ABTS^{\bullet-}$ solution was immediately used to perform the aforementioned experiment.

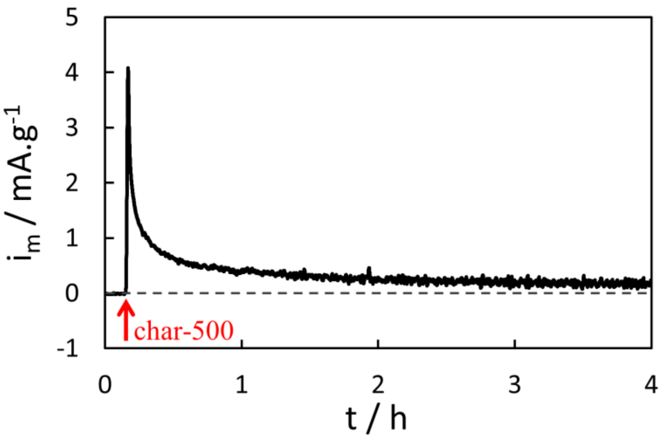

Supplementary Figure S13. Evolution of the current which would be recorded per gram of char-500 introduced in a system as used by Klüpfel et al. ("mediated electrochemical oxidation").[7] These data comes from the derivative with respect to *t* over 4 h of our continuous measurement such as presented in Figure S18, i.e. with 50 mM ferricyanide. The shape and the initial peak current magnitude are very similar to the one recorded in their study (after recalculation with respect to the mass of char introduced). After 1 or 2 h, the rate of electron donation became so low (and its decrease so slow) that the current recorded could easily be mistaken as a single baseline current. In any case, integrating a similar current over a long time to obtain an accurate EDC would be challenging, even more in the presence of a baseline current of similar or higher magnitude.

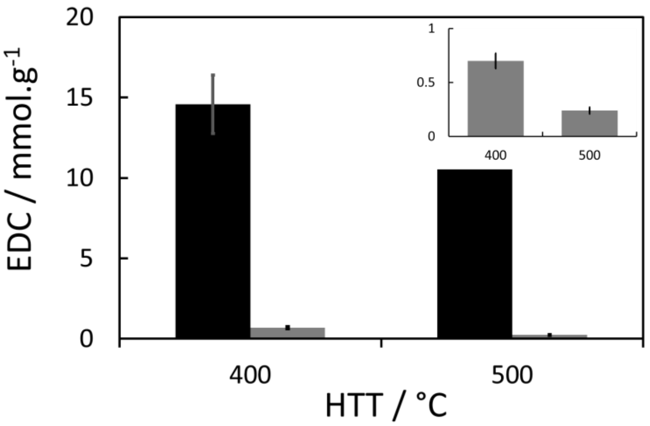

Supplementary Figure S14. EDC (top) of grass-based biochars pyrolysed at HTT of 400 °C and 500 °C. Our data (black bars, t = 30 d) are compared with those of Klüpfel *et al.* (grey bars, with a zoom in inset for their EDC, n = 3). Error bars represent 2 standard deviations (n = 2 for grass-400 and n = 1 for grass-500).

It must be noted that Klüpfel *et al.* used as feedstock the grass specie *Festuca arundinacea* while we used *Panicum virgatum*. The key parameters (elemental composition, specific surface area, etc.) of biochars resulting from similar feedstock (e.g. wood or grass whatever the specie – e.g. poplar, pine or oak for wood) were similar when the HTT was identical[8]. Therefore the use a different grass species (from the same family: *Poaceae*) is not expected to imply such a large difference in EDC and time of reaction.

Note also that in both studies the EDC of grass-based biochars are substantially higher than the wood-based biochar of identical HTT.

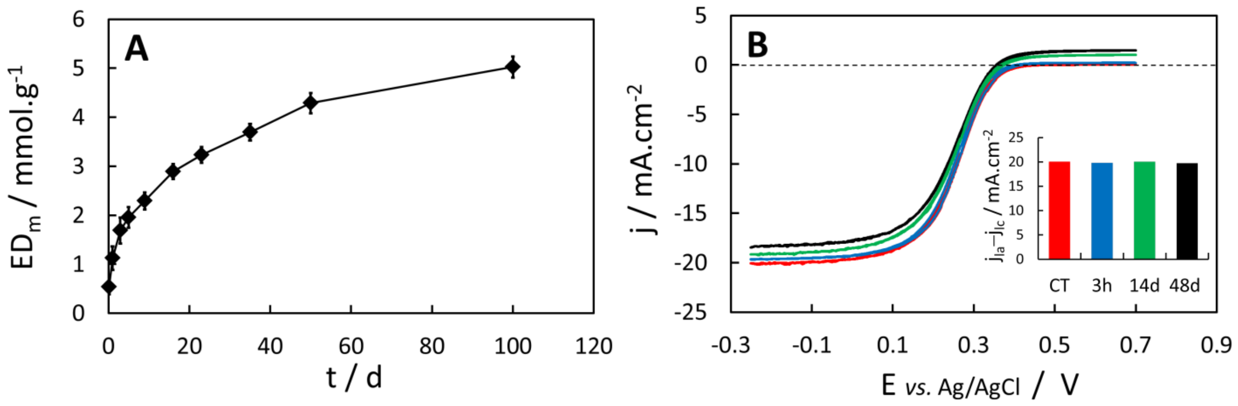

Supplementary Figure S15. (A) Evolution of the ED_m of a synthetic humic acid (Aldrich) solution/suspension, n =3. (**B**) CVs recorded for the control devoid of humic acid (at t = 0, red) and for one suspension of humic acid (0.95 g.L^{-1}) at t = 3 h (blue), t = 14 d (green) and t = 48 d (black); recorded at 50 mV.s^{-1}, 1000 rpm. Inset shows the corresponding differences between anodic and cathodic plateau current densities (j_{la}–j_{lc}). The conservation of this difference shows that (i) no significant amount of ferricyanide or ferrocyanide was adsorbed on the solid fraction of the humic acid; and (ii) ferricyanide and ferrocyanide were chemically stable even in the presence of humic acid.

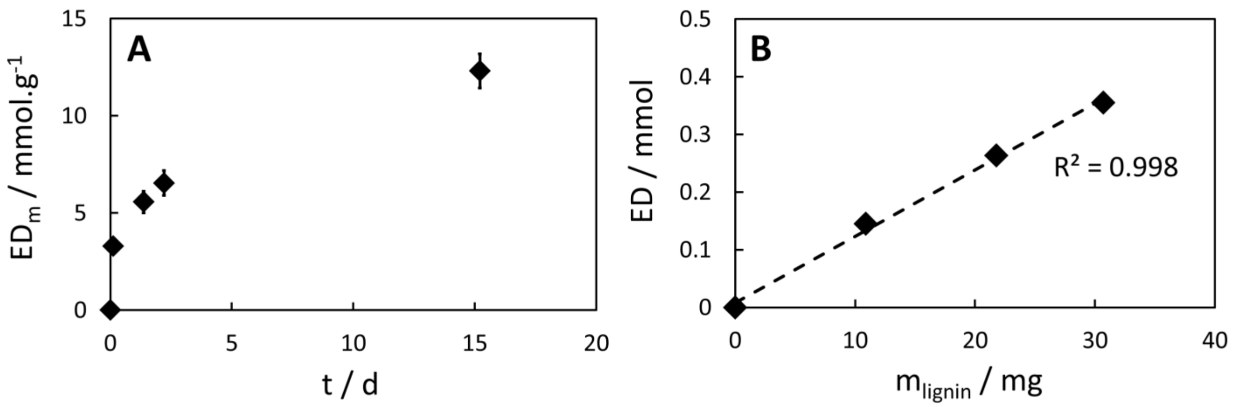

Supplementary Figure S16. (**A**) Evolution of the ED_m of a synthetic lignin powder (Aldrich) solution/suspension (n = 3). (**B**) linearity of the amount of electrons donated with the mass of lignin initially introduced. As for biochars, the EDC value provided at the end of the experiments (t = 15 d) is likely underestimated.

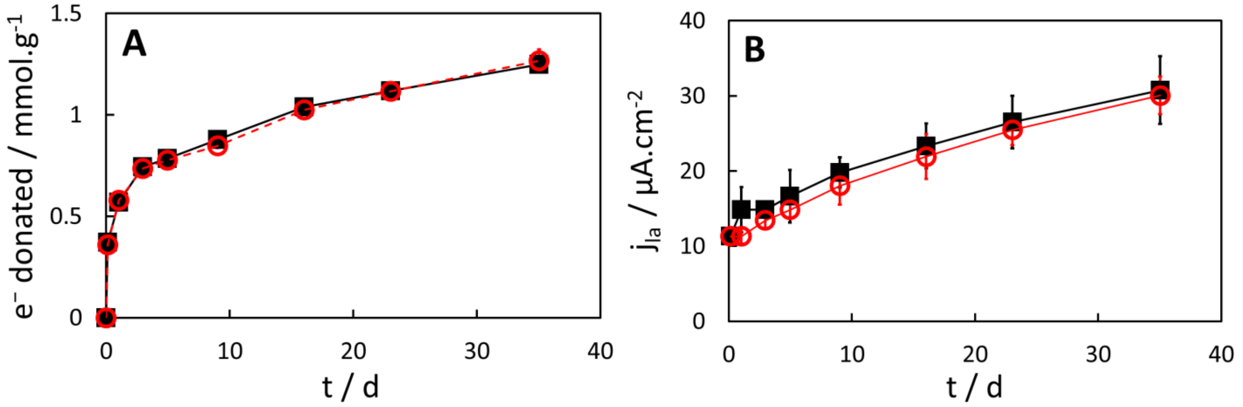

Supplementary Figure S17. (**A**) Evolution of the charge donated by char-600 in the presence (red circles) or the absence (black squares) of surfactant (1 mM CTAB). Increasing the wettability of the char did not increase the kinetics of electron donation. (**B**) Evolution of j_{la} for the corresponding controls (without char): the surfactant did not impact the very slow reduction of ferricyanide. For the sake of comparison, final j_{la} in the presence of ~ 30 mg of char-600 (t = 35 d) was typically ~ 340 µA.cm^{-2}. Error bars represent 2 standard deviations for 2 samples (mostly not visible in (**A**)).

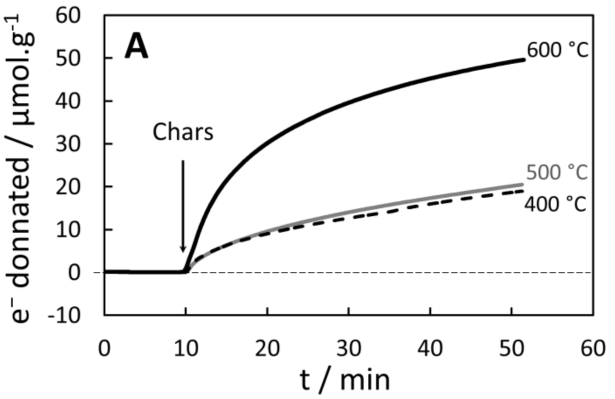

Supplementary Figure S18. (**A**) Real-time monitoring of electron donation from the chars of different HTTs to the ferricyanide solution. Measured via chronoamperometry at + 0.7 V vs. Ag/AgCl and 1000 rpm, with a recording period of 1 s. Solution: 10 mM ferricyanide, 0.4 M NaCl, 0.1 M phosphate buffer pH 6.5. Grinded chars (< 100 µm) were added to the solution at t = 10 min. Note that these kinetics must be somehow slightly underestimated since, in this case, a fraction of the rather hydrophobic chars remained at air/solution/solids interfaces despite the solution stirring by the RDE (char-600 being the most hydrophobic). Note also a difference in the concentrations of ferricyanide and NaCl with respect to the other experiments of the present study. This difference does not call into question the conclusion of the present results (faster initial kinetics of electron donation for char-600). Non-exclusive hypotheses for this faster initial kinetics of electron donation from the char of smaller EDC are:

(1) an easier/faster access for ferricyanide trianion to the oxidizable moieties present in the pores of the char-600 than in the less accessible, more negatively-charged pores of lower HTT chars (see core text);
(2) a delocalization of a fraction of the redox reactions since the HTT may be high enough to produce a slightly conductive char owing to the formation and alignment in sheets of conjugated aromatic carbon[9-11].

Supplementary Method S3: solution for EAC determination. The reducing solution was made of ~ 5 mM reduced neutral red (NR), 3 M NaCl and 0.1 M PB (pH 6.5). NR was reduced by electrolysis in the anaerobic chamber at 30 °C in the cathodic chamber of a custom made 2-compartment cell[12] wrapped under aluminum foil to avoid light exposure. An anion exchange membrane (AMI-7001, Membranes International, Ringwood, USA) separated the cathodic and anodic compartment (a platinum wire was used as counter electrode in the 1 M $NaNO_3$ anolyte). The reduction was performed by chronoamperometry at – 0.65 V *vs.* Ag/AgCl on a carbon felt (Alfa Aesar/VWR, 3.18 mm thickness) working electrode (projected surface area of ~ 50 cm^2) previously treated with CTAB surfactant to enhance hydrophilicity as described elsewhere[13].

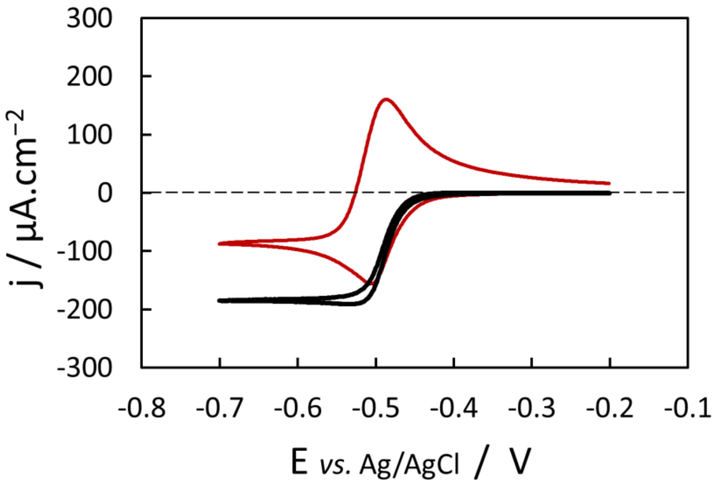

Supplementary Figure S19. Stagnant (red) and hydrodynamic (black, 1000 rpm) CVs of 0.3 mM NR solution, recorded at 20 $mV.s^{-1}$. Peak to peak difference of the stagnant CV was ~ 25 mV, close to the theoretical value for a reversible 2-electron transfer[14]. Hydrodynamic CV shows that – 0.65 V vs. Ag/AgCl is a sufficiently low potential to monitor the cathodic limiting current j_{lc} without generating any significant H_2 evolution.

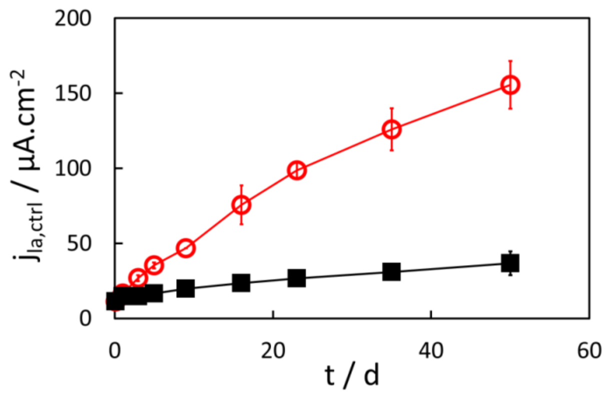

Supplementary Figure S20. Evolution of $j_{la,ctrl}$ for the 50 mM ferricyanide electrolyte under dark (black squares) and under natural light cycle (red circles). For the sake of comparison, current densities recorded in the presence of ~ 30 mg of chars ranged from ~ 340 µA.cm^{-2} for char-600 to ~ 1300 µA.cm^{-2} for char-400 after 50 days of reaction. Error bars represent 2 standard deviations (n = 2).

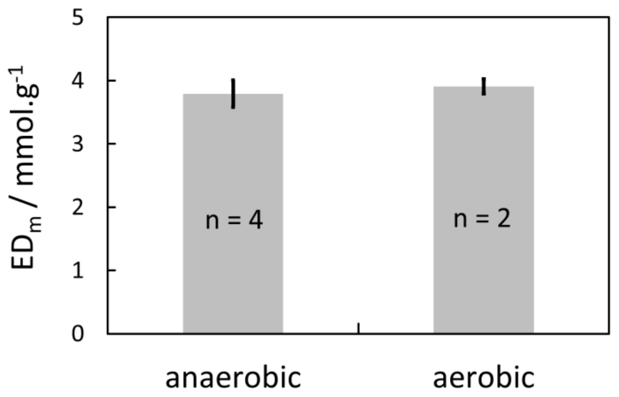

Supplementary Figure S21. Impact of initial oxygen removal in recorded EDC values. EDCs recorded after 12 days of reaction of char-400 with the ferricyanide solution, either with (‘anaerobic’) or without (‘aerobic’) initial degasing of dry char (15 h in vacuum) and ferricyanide solution. Error bars represent 2 standard deviations for n samples. The absence of substantial impact was rather expected since ferrocyanide solutions are known to be kinetically stable even under aerobic conditions when conserved under dark, at mild T and circumneutral pH[15].

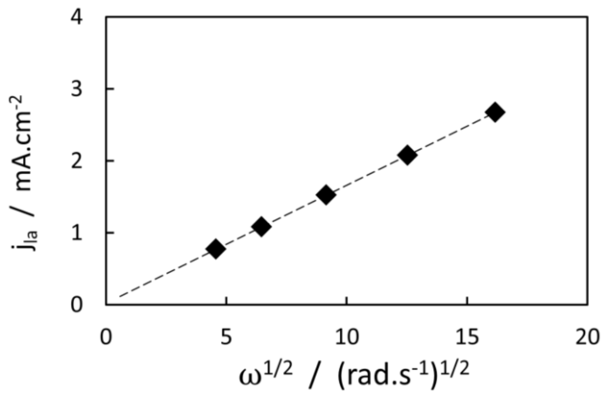

Supplementary Figure S22. Levich plot recorded in a solution of 5 mM potassium ferrocyanide with 0.1 M sodium phosphate buffer and 3 M NaCl. Recorded in anaerobic condition at 30 °C and + 0.7 V vs. Ag/AgCl. The slope value derived with the Levich equation provides D_{ferro} at 4.27 ± 0.28 $cm^2.s^{-1}$ (n = 3).

Supplementary References:

1 Haynes, C. G., Turner, A. H. & Waters, W. A. The oxidation of monohydric phenols by alkaline ferricyanide. *J. Chem. Soc.*, 2823-2831 (1956).

2 McDonald, P. D. & Hamilton, G. A. in *Organic Chemistry* Vol. Volume 5, Part B 97-134 (Elsevier, 1973).

3 Song, Y. *et al.* ABTS as an Electron Shuttle to Enhance the Oxidation Kinetics of Substituted Phenols by Aqueous Permanganate. *Environ. Sci. Technol.* **49**, 11764-11771 (2015).

4 Isono, T. Density, viscosity, and electrolytic conductivity of concentrated aqueous electrolyte solutions at several temperatures. Alkaline-earth chlorides, lanthanum chloride, sodium chloride, sodium nitrate, sodium bromide, potassium nitrate, potassium bromide, and cadmium nitrate. *J. Chem. Eng. Data* **29**, 45-52 (1984).

5 Kestin, J., Khalifa, H. E. & Correia, R. J. Tables of the dynamic and kinematic viscosity of aqueous NaCl solutions in the temperature range 20–150 °C and the pressure range 0.1–35 MPa. *J. Phys. Chem. Ref. Data* **10**, 71-88 (1981).

6 Sander, R. Compilation of Henry's law constants (version 4.0) for water as solvent. *Atmos. Chem. Phys.* **15**, 4399-4981 (2015).

7 Klüpfel, L., Keiluweit, M., Kleber, M. & Sander, M. Redox Properties of Plant Biomass-Derived Black Carbon (Biochar). *Environ. Sci. Technol.* **48**, 5601-5611 (2014).

8 Ahmad, M. *et al.* Biochar as a sorbent for contaminant management in soil and water: A review. *Chemosphere* **99**, 19-33 (2014).

9 Nishimiya, K. Analysis of chemical structure of wood charcoal by X-ray photoelectron spectroscopy. *J. Wood Sci.* **44**, 56-61 (1998).

10 Cao, X. *et al.* Characterization of Wood Chars Produced at Different Temperatures Using Advanced Solid-State 13C NMR Spectroscopic Techniques. *Energy Fuels* **26**, 5983-5991 (2012).

11 Lehmann, J. & Joseph, S. *Biochar for Environmental Management : Science, Technology and Implementation (2nd ed.)* (Routledge, Abingdon, UK, 2015).

12 Guo, K. *et al.* Flame Oxidation of Stainless Steel Felt Enhances Anodic Biofilm Formation and Current Output in Bioelectrochemical Systems. *Environ. Sci. Technol.* **48**, 7151-7156 (2014).

13 Guo, K. *et al.* Surfactant treatment of carbon felt enhances anodic microbial electrocatalysis in bioelectrochemical systems. *Electrochem. Commun.* **39**, 1-4 (2014).

14 Bard, A. J. & Faulkner, L. R. *Electrochemical Methods: Fundamentals and Applications (2nd ed.)* (Wiley, New York, 2001).

15 Asperger, S. Kinetics of the decomposition of potassium ferrocyanide in ultra-violet light. *Transactions of the Faraday Society* **48**, 617-624 (1952).
